# Body-maps of emotions in bilateral vestibulopathy

**DOI:** 10.1007/s00415-020-09888-z

**Published:** 2020-06-17

**Authors:** Estelle Nakul, Charles Dabard, Michel Toupet, Charlotte Hautefort, Christian van Nechel, Bigna Lenggenhager, Christophe Lopez

**Affiliations:** 1 Laboratoire de Neurosciences Sensorielles et Cognitives (LNSC) UMR 7260, Centre National de la Recherche Scientifique (CNRS), Centre Saint-Charles, Fédération de Recherche 3C-Case B, Aix Marseille Univ, 3, Place Victor Hugo, 13331 Marseille Cedex 03, France; 2 Institut de Recherche en Oto-Neurologie, IRON, Paris, France; 3 Centre d’Explorations Fonctionnelles Oto-Neurologiques, Paris, France; 4 Service ORL, Hôpital Lariboisière, AP-HP, Paris, France; 5 Unité Troubles de L’Equilibre and Vertiges, CHU Brugmann, Brussels, Belgium; 6 Unité de Neuro-Ophtalmologie, CHU Erasme, Brussels, Belgium; 7 Clinique des Vertiges, Brussels, Belgium; 8 Cognitive Neuropsychology, Department of Psychology, University of Zurich, Zurich, Switzerland

Patients with a bilateral vestibulopathy (BVP) report a poorer quality of life, in both its physical and social dimensions, than healthy control participants [1]. BVP patients may report spatial anxiety with deficits in spatial cognition [2], and abnormal autonomous regulation [3]. Yet, they have lower anxiety related to vertigo [4] and less psychiatric comorbidities [5] than most patients with episodic vertigo. How BVP patients experience emotions—other than anxiety and depression—in an embodied way, is poorly documented, despite evidence of tight connections between vestibular, emotional, and embodiment processes [6]. Here, we investigated the embodiment of emotions in BVP patients. Emotions are strongly embodied: happiness, sadness and fear, for example, are associated with a variety of physiological changes [7] involving the interoceptive and somatosensory systems [8]. We used the computerized emBODY tool [9, 10] to document body-maps of emotions in BVP patients and healthy controls. Experiments with the emBODY tool in large samples of healthy participants revealed that different emotions are consistently associated with different body-maps [9, 10]. These body-maps of emotions are consistent across different cultures [9], developed between age 6 and 17 to become spatially specific [11], and they appear to be altered in clinical conditions [12].

**Fig. 1:**
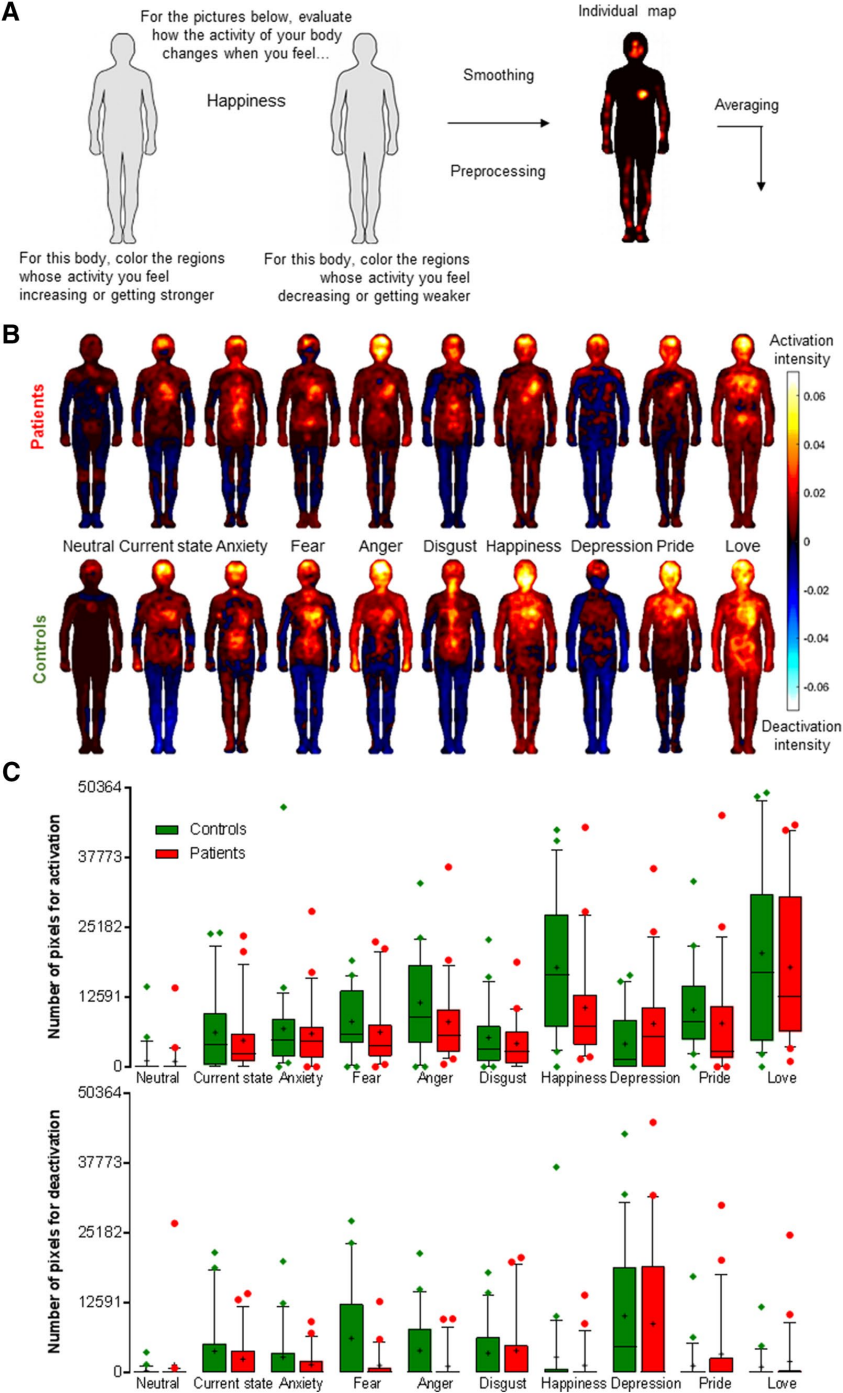
Procedures for the emBODY tool and results. **a** A 2D abstract human silhouette on the left side of the computer screen was to report activated body parts, and a silhouette on the right was to report deactivated body parts. Participants colored silhouettes using a mouse in their dominant hand. The coloring was dynamic and there was no time limit to complete the task. The coloring tool had a 12-pixel diameter and successive clicks on a region increased the color opacity, to represent the intensity of bodily sensations associated with an emotion. Silhouettes were simple, without indication of internal organs, to help participants concentrate on the sensations and their spatial distribution, rather to suggest conceptual associations between organs and emotions [9]. Pre-processing and statistical analyses were adapted from Refs. [9, 12]. Silhouettes contained 50,364 pixels for which the color intensity was coded from 0 to 100. As a single mouse click would color several hundred pixels, we accounted for spatial dependencies using a Gaussian disk to smooth maps data and to prevent overstatement of embodiment. Values for each pixel and each emotion from both silhouettes were computed in a single figure to obtain individual combined maps as showed in the right part of the figure. Activation and deactivation maps were also kept separately. Individual maps were averaged to generate mean body-maps of emotions represented in part B. **b** Mean body-maps of emotions for patients (upper panel) and controls (bottom panel). Warm and cold colors correspond to body activation and deactivation respectively. Color code from − 1 (maximum deactivation) to 1 (maximum activation). **c** Number of pixels colored for activation and deactivation. Box and whiskers plots show medians (horizontal line) and interquartile ranges (10–90). Means are shown as + and dots are outliers

We tested 21 BVP patients and 21 healthy controls matched for age, sex, education, and body-mass index (Table 1). Anxiety and depression [13] did not differ between groups. Standard otoneurological examinations established that patients presented a severe bilateral vestibular hypofunction without neurological disorder (Table 1). Controls reported no history of otoneurological disorder. Participants reported the bodily sensations associated with a neutral state, their current state, four basic emotions (fear, anger, disgust, happiness), and four non-basic emotions (anxiety, love, depression, pride). Each emotional word was presented once in a randomized order. Participants indicated *where* exactly in their body and *how intensely* they perceived an increase or decrease in activation when experiencing that emotion by coloring 2D human silhouettes on a computer screen (see Fig. 1a for details on procedures). Increase or decrease in activation was meant to reflect changes in interoceptive signals, such as higher/lower heart and breathing rates, palm sweating, skin temperature, or tonus in the legs and facial musculature. Thus, all sensed and reported changes in such inner bodily signals (no matter if the feeling is of activation or deactivation) would correspond to high interoception (i.e., awareness of inner bodily states).

**Table 1:** Demographic and clinical characteristics of BVL patients and healthy controls

	Patients	Controls	Statistics
Males/females (*n*)	8/13	8/13	–
Age (years)	62 ± 13	62 ± 12	*t* = − 0.40, *p* = 0.69
Education (*n*)			
Level 1	3	1	*χ*^2^ = 8.4, *p* = 0.08
Level 2	0	2	
Level 3	3	3	
Level 4	8	2	
Level 5	7	13	
Body-mass index	24 ± 4	24 ± 3	*t* = − 0.06, *p* = 0.96
Anxiety score	8.5 ± 2.6	7.3 ± 3.2	*t* = − 0.52, *p* = 0.60
Depression score	4.3 ± 2.3	4.0 ± 2.7	*t* = 0.50, *p* = 0.62
Caloric test (mean slow phase eye velocity in °/s)			
Left ear at 44 °C	0.92 ± 1.91	–	–
Right ear at 44 °C	1.76 ± 2.76		
Left ear at 30 °C	2.16 ± 2.92		
Right ear at 30 °C	2.14 ± 5.84		
vHIT (gain)			
Horizontal canals	0.32 ± 0.28	–	–
Anterior canals Posterior canals	0.22 ± 0.25 0.25 ± 0.27		
cVEMPs (μV)			
Left ear	121 ± 23 (*n* = 6)	–	–
Right ear	73 ± 51 (*n* = 9)		
oVEMPs (μV)			
Left ear	2.0 ± 1.8 (*n* = 3)	–	–
Right ear	2.7 ± 0.48 (*n* = 3)		

Education level according to the French education system; Level 1: before high school; Level 2: accomplished high school; Level 3: 2 years after high school; Level 4: Bachelor’s degree, Level 5: Master’s degree, Engineering degree, PhD, MD. Levels of anxiety and depression the week before the experiment were reported using the Hospital Anxiety and Depression scale [13]. All patients had weak responses to a bithermal caloric test with water at 44 °C and 30 °C (mean slow phase eye velocity < 5°/s) and reduced responses to the video head impulse test (vHIT) with a mean gain < 0.7. The saccular and utricular functions were evaluated by cervical vestibular-evoked myogenic potentials (cVEMPs) over the sternocleidomastoid muscles and ocular vestibular-evoked myogenic potentials (oVEMPs) over the inferior oblique muscles. The VEMP amplitude is reported only for the patients with VEMPs (number in brackets). All data are reported as mean ± SD

First, individual maps were averaged to generate group body-maps representing the mean intensity of reported emotions for patients and controls [9, 10] (Fig. 1b). Visual inspection of group body-maps showed lower activation for positive emotions (e.g., happiness, pride, love), and lower deactivation for negative emotions (e.g., fear, anger, disgust, depression) in patients compared to controls. However, for none of the emotions, Mann–Whitney *U* tests revealed a significant difference between patients and controls after correcting for multiple comparisons.

Second, we analyzed the number of colored pixels corresponding to activation and deactivation for each emotional word per participant [12] (Fig. 1c). Friedman’s ANOVA revealed a significant effect of emotions on the number of colored pixels in patients (*χ*^2^ = 82.60, *p* < 0.001) as well as in controls (*χ*^2^ = 83.63, *p* < 0.001). Thus, as expected, the type of emotion significantly modulated the extent of the reported activation for both groups. Mann–Whitney *U* tests indicated that patients colored significantly less pixels for activation for happiness, when compared to controls (*U* = 135.0, *Z* = − 2.15, *p* = 0.03), whereas there was no difference for the other emotions and the current state (all *U* < 219.5, *Z* < − 1.82, *p* > 0.07). By contrast, Friedman’s ANOVA revealed no effect of emotions for deactivation maps in patients (*χ*^2^ = 15.82, *p* = 0.07), whereas the effect of emotions was significant in controls (*χ*^2^ = 29.39, *p* = 0.001). This indicates a lack of emotion-specific response for BVP patients. Mann–Whitney *U* tests indicated no difference in the number of pixels colored for deactivation between patients and controls for all emotions (all *U* < 220.5, *Z* < 451.5, *p* > 0.10).

Our exploratory study suggests that, overall, the topography of bodily sensations was not significantly different in BVP patients  and in controls. Yet, a limitation is that vestibular function was not tested in the controls. The task is mainly based on the recall of bodily sensations typically experienced during one specific emotion. The lack of clear topographical difference between BVP patients and controls suggests that this type of memory is not altered in severe long-term bilateral vestibular hypofunction. This is in contrast with deficits in spatial memory found in BVP (e.g., navigation in a virtual Morris water maze [14]). Functional MRI [15] revealed that basic emotions were best classified by activity patterns in structures along the brain midline (medial prefrontal cortex, posterior cingulate cortex, precuneus), in the frontal pole and sensorimotor regions (post- and precentral gyri, insula). Midline structures, involved in self processing, are likely to integrate interoceptive signals with representations from the self and memory [15]. Thus, BVP may have a weaker impact on these midline structures than on the neural networks underpinning spatial memory [2, 14], sparing the topography of bodily sensations, while disrupting extrapersonal space memory.

When considering the number of colored pixels, we found a significantly reduced activation for happiness in the patients. This may reflect a form of emotional numbing, a symptom belonging to depersonalization–derealization, shown to be more frequent and more intense in patients with peripheral vestibular disorders [16]. Importantly, unlike healthy controls, BVP patients showed no emotion-specific modulation of the extent of the deactivation response. This difference cannot be accounted for by the participants’ age, body-mass index, anxiety, and depression scores. This suggests that BVP patients may be less aware of their interoceptive signals, i.e., the signals from the gastro-intestinal tract, blood vessels, kidneys, heart, etc., when presented with different emotional words. The emBODY tool revealed abnormal, less clear, and less differentiated body-maps of emotions in patients with schizophrenia [12], known to have lower interoceptive accuracy and higher disembodiment than healthy controls [17]. There is evidence that anosmia [18] and sensorimotor deafferentation in spinal cord injury [19] can decrease interoceptive accuracy. Similarly, BVP may alter interoceptive accuracy and attenuate the emotion-specific modulation of the spatial extent of bodily sensations, possibly due to the strong connections between the vestibular and interoceptive signals at both anatomical and functional levels [20].

In our sample, the general anxiety and depression scores (as measured with the Hospital Anxiety and Depression Scale [13]) did not differ between patients and controls. It has previously been argued that such findings might reflect a reduced anxiety, even though BVP patients encounter objectively more dangerous situations (e.g., they fall more often) [4, 21]. This is further confirmed by the fact that BVP patients show significantly lower anxiety scores and less psychiatric comorbidities in comparison to most patients with episodic vestibular disorders and chronic functional dizziness [4, 5, 21]. However, higher salivary cortisol levels have been found in BVP patients compared to healthy controls [3], indicating that physiological markers of anxiety might be increased, although not reflected in subjective reports. We tentatively suggest that the reduced sensitivity to emotionally induced changes in deactivations found in our study might corroborate such findings. However, more evidence will be needed to establish such effect, especially as our finding was specific to perceived deactivation.

In conclusion, although embodiment of emotions should be assessed in larger samples of participants with vestibular disorders, we believe that the present work helps understand the complex symptomatology of BVP patients, which needs to be better characterized, especially regarding own-body, self and emotional processing.
